# Comparative measurement of aortic root by transthoracic echocardiography in normal Korean population based on two different guidelines

**DOI:** 10.1186/1476-7120-11-28

**Published:** 2013-08-13

**Authors:** Myoung Kyun Son, Sung-A Chang, Ji Hye Kwak, Hye Jin Lim, Sung-Ji Park, Jin-Oh Choi, Sang-Chol Lee, Seung Woo Park, Duk-Kyung Kim, Jae K Oh

**Affiliations:** 1 Division of Cardiology, Department of Medicine, Cardiovascular Imaging Center, Samsung Medical Center, Sungkyunkwan University School of Medicine, Seoul, Republic of Korea; 2 Division of Cardiovascular Diseases, Mayo Clinic College of Medicine, Rochester, Minnesota, USA

**Keywords:** Aortic root measurement, Normal reference, Transthoracic echocardiography

## Abstract

**Background:**

Aortic root size is an important parameter in vascular diseases and can be easily assessed by transthoracic echocardiography. However, measurements values may vary according to cardiac cycle and the definition used for edge. This study aimed to define normal values according to the measurement method specified by two different guidelines to determine the influence of the different methods on echocardiographic measurements.

**Methods:**

Healthy Korean adults were enrolled. The aortic root diameters were measured twice at four levels (aortic annulus, sinuses of Valsalva, sinotubular junction, and ascending aorta) by the 2005 American Society of Echocardiography (ASE) guidelines (measured from leading edge to leading edge during diastole) and the 2010 ASE pediatric guidelines (measured from inner edge to inner edge during systole).

**Results:**

One hundred twelve subjects aged 20–69 years were enrolled. The aortic diameters (cm) determine by the aforementioned two guidelines showed significant difference. Measurements were larger in 2005 ASE guideline at aortic annuls, sinuses of Valsalva, and sinotubular junction level, but smaller at ascending aortic level with 2-3mm of differences. Intraobserver variability was similarly good, but interobserver variability was slightly higher than intraobserver variability in both measurement methods. BSA and age was most important determinant for aortic root size.

**Conclusions:**

The measurement method of aortic root can affect the echocardiographic result. The measurement method should be noted when assessing clinical significance of aortic root measurement.

## Background

Measurement of aortic diameter is important for diagnosis and monitoring of vascular diseases [1-3]. For instance, diagnostic criteria of Marfan syndrome include aortic root dilation and change of aortic root size is a marker for aortic complications and indication of surgery [1,3,4]. It has also been suggested that aortic root dilation may be associated with higher cardiovascular morbidity and mortality in subjects without previous cardiovascular disease [5,6]. Transthoracic echocardiography (TTE) can easily access the aortic root and repeated measurements are safe and reliable [7,8]. Several guidelines have suggested specified measurement methods to improve the reliability and validity of aortic root measurements with TTE [2,9-11]. However, aortic root size may change according to cardiac cycle and definition for edge, and variable results of aortic root size can be reported in the same subject [2,11].

Recently, the American Society of Echocardiography (ASE) introduced a new pediatric guideline that specifies measurement method from the inner edge to the inner edge during systole [2], which differs from the previous ASE chamber quantification guidelines in adults [11]. Because patients with vascular disease frequently develop their disease from childhood [1,4], different measurement methods could affect measurement result when patients move from pediatric to adult clinic. The aim of this study was to define normal values of aortic root diameters according to the measurement method based on the two different guidelines and to determine if their measurement values and associations with clinical characteristics are influenced by measurement methods.

## Methods

### Study population

Clinically normal Korean adults (20–69 years, n=112) were prospectively recruited. All subjects were asymptomatic and had no history of cardiovascular disease. Exclusion criteria were history of hypertension or antihypertensive treatment, history of diabetes mellitus, end-stage renal disease, and other acute or chronic systemic diseases. Informed consent was obtained from each participant. Our institutional review board approved the study. Body surface area (BSA) was computed using the Dubois and Dubois formula [12]: BSA (m^2^) = 0.007184 × height (cm)^0.725^ × weight (kg)^0.425^.

### Echocardiography

**Figure 1:**
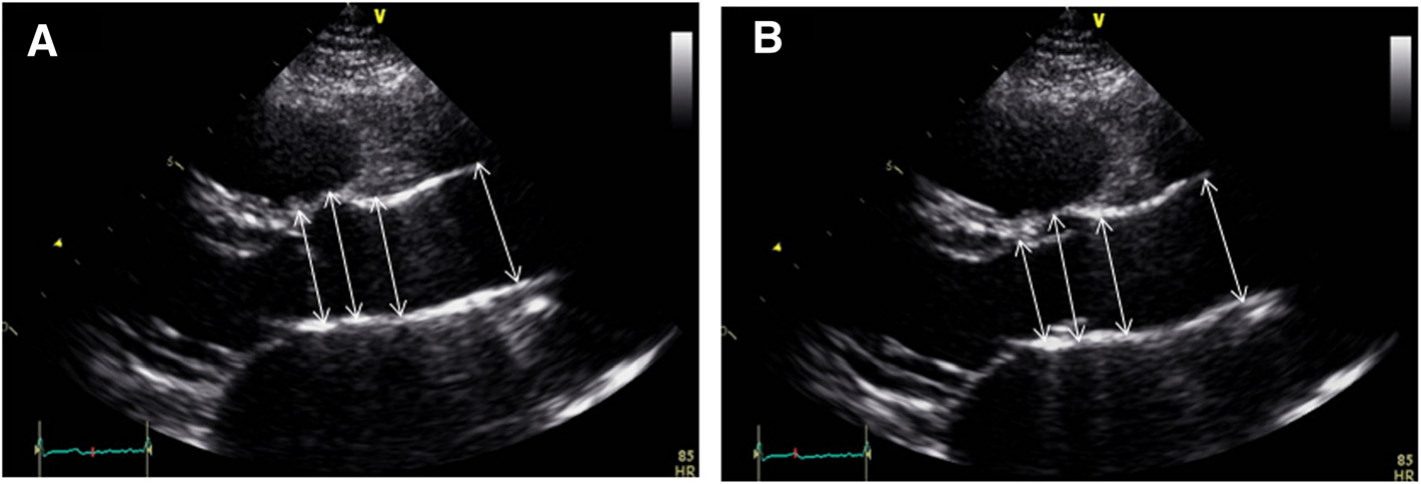
Representative case of measurement of aortic diameter by (A) 2005 American Society of Echocardiography (2005 ASE) chamber quantification guidelines and (B) 2010 ASE pediatric measurement guidelines (2010 Pediatric).

TTE was performed using a Vivid 7 instrument (GE Medical Systems, Horten, Norway) with subjects in the left lateral decubitus position. The subjects were required to rest for 5 minutes before examination. All examinations were carried out by a single experienced sonographer who was blinded to the clinical data. Image was acquired by single sonographer and measurement was performed after closing of exam by two sonographers. The aortic root diameters were measured at four levels (aortic annulus, sinuses of Valsalva, sinotubular junction, and ascending aorta) with the use of a parasternal long-axis view. One measurement was performed according to the 2005 ASE chamber quantification guidelines (Figure 1A) [11]. The aortic root diameters were measured in diastole, perpendicular to the long axis of the aorta, using the leading edge to leading edge technique. Thus, our measurements included the anterior wall of the aorta and not the posterior wall. The second measurement was obtained according to the 2010 ASE pediatric measurements guidelines (Figure 1B) [2]. The maximal aortic diameter was measured from the inner edge to inner edge of the aortic wall during ventricular systole on an axis perpendicular to the path of blood flow.

To assess intraobserver and interobserver variability, two experienced sonographers measured aortic root diameter of randomly selected 20 subjects.

### Statistical analysis

Continuous variables were described as mean ± SD. Categorical variables were expressed as a number and as a percentage (%). One-way analysis of variances (ANOVA) was used to compare the differences among age groups. Paired t-test was performed to compare the measurement result of aortic root parameters using two different guidelines.

Correlations between clinical characteristics and aortic root diameters obtained with two different guidelines were analyzed using multiple linear regression analysis. Sex was coded as dummy variable (male, 1; female, 0). DBP was input to regression models because it showed higher correlation with aortic diameters than SBP did in all the levels of aortic root. SBP was not adopted as the models for preventing collinearity because of higher correlation between SBP and DBP (r=0.901). A z score was also calculated for each aortic root measurement with standard algorithms. The z score meant the standard deviation (SD) from the mean aortic diameter normalized for the subject’s BSA and age [9,13,14].

Intraclass correlation coefficient (ICC) was used as a measure of the magnitude of reliability agreement. The ICC is the proportion of the variability of different ratings of the same subject to the total variation across all ratings for all subjects [15,16]. The ICC is large (i.e., ~1) when there is little variance. A *P* < 0.05 was considered to be statistically significant. Statistical analysis was performed using the Statistical Package for the Social Sciences (SPSS) version 18.0 for Windows.

## Results

**Figure 2:**
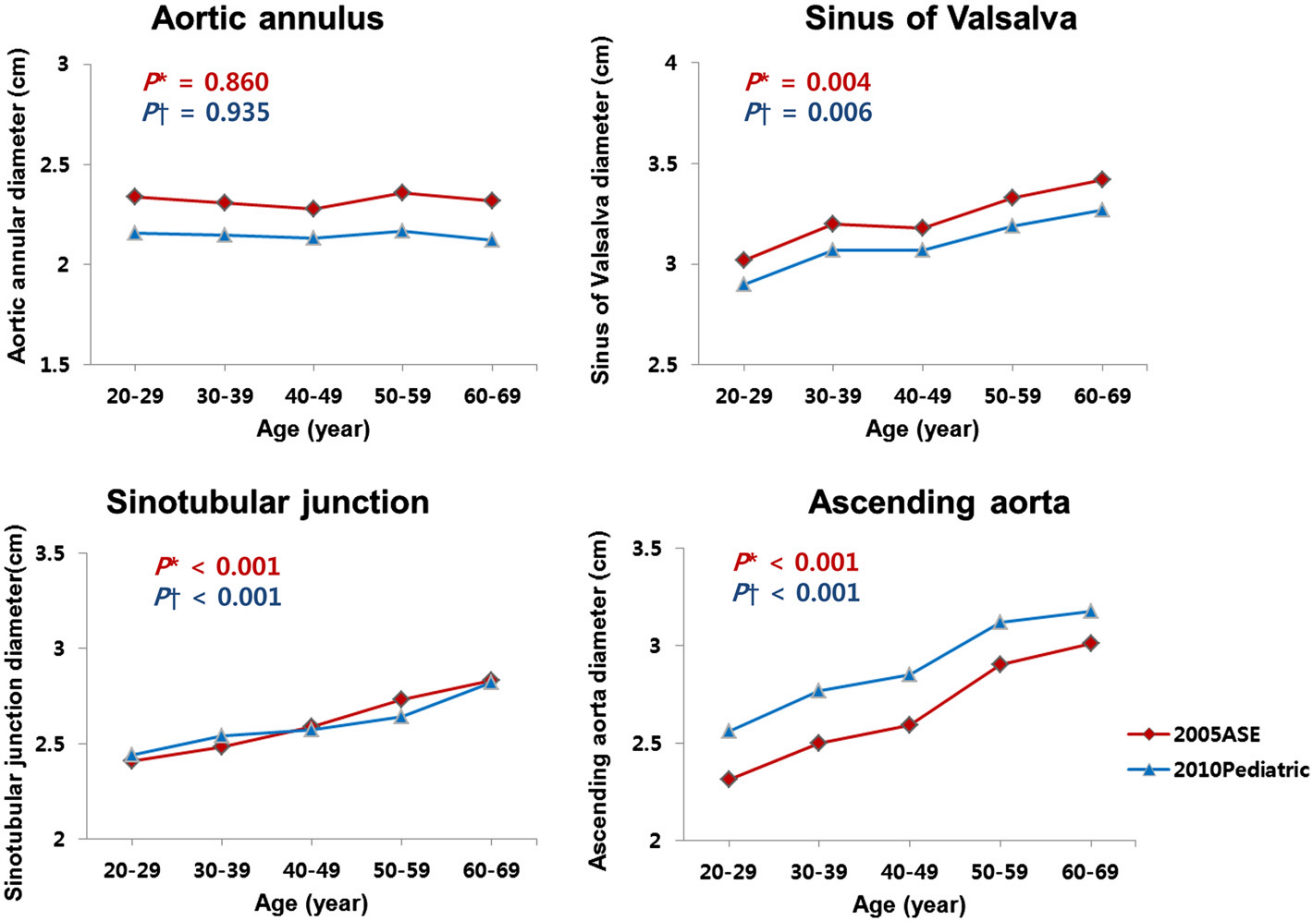
Mean aortic diameters measured by 2005 American Society of Echocardiography (2005 ASE) chamber quantification guidelines and 2010 ASE pediatric measurement guidelines (2010 Pediatric) according to age groups (*P values were obtained by ANOVA for differences of mean aortic diameters measured by 2005 ASE chamber quantification guidelines according to age groups; † P values were obtained by ANOVA for differences of mean aortic diameters measured by 2010 ASE pediatric measurement guidelines according to age groups).

Our study population consisted of 60 men and 52 women, aged 20–69 years (mean 44.2 ± 13.1 years). Clinical characteristics according to age groups are presented in Table 1. Systolic blood pressure (SBP) and diastolic blood pressure (DBP) had a tendency to increase, and BSA decreased as age increased. The mean aortic diameters (cm) determined by the aforementioned 2005 and 2010 pediatric guidelines were: aortic annulus, 2.33 ± 0.24 and 2.15 ± 0.21; sinuses of Valsalva, 3.24 ± 0.38 and 3.11 ± 0.35; sinotubular junction, 2.61 ± 0.32 and 2.60 ± 0.29; and ascending aorta, 2.67 ± 0.41 and 2.91 ± 0.37, respectively. The mean aortic diameters obtained by the 2005 ASE guidelines and 2010 ASE pediatric guidelines according to age groups are presented in Table 2. The diameters of sinuses of Valsalva, sinotubular junction, and ascending aorta increased with age. The differences of these diameters according to age groups were statistically significant by use of ANOVA (Figure 2). The diameters of aortic annulus, sinuses of Valsalva, sinotubular junction were larger, but that of ascending aorta was smaller when measured by the 2005 ASE guidelines than by the 2010 ASE pediatric guidelines. The mean differences between these diameters measured by 2005 ASE guidelines and 2010 ASE pediatric guidelines were statistically significant (Table 2).

**Table 1:** Clinical characteristics stratified by age

**Age group (yr)**	**20-29**	**30-39**	**40-49**	**50-59**	**60-69**
**M/F**	**(12/10)**	**(14/9)**	**(10/8)**	**(14/14)**	**(10/11)**
No.	22	23	18	28	21
HR, beats/min	71 ± 13	70 ± 9	69 ± 9	67 ± 12	69 ± 11
SBP, mmHg	117 ± 12	120 ± 17	121 ± 12	130 ± 18	130 ± 16
DBP, mmHg	70 ± 8	73 ± 13	75 ± 10	81 ± 12	79 ± 10
BSA, kg/m^2^	1.76 ± 0.21	1.74 ± 0.21	1.73 ± 0.15	1.68 ± 0.15	1.67 ± 0.15

*BSA* body surface area, *DBP* diastolic blood pressure, *HR* heart rate, *SBP* systolic blood pressure.

Data are expressed as mean ± SD or as number.

**Table 2:** Aortic diameters measured by two different echocardiography guidelines according to age groups

	**2005 ASE** ^*^	**2010 Pediatric** ^**†**^	**Difference**		
			**Mean**	**SD**	***P ***^**‡**^
20-29 yr					
Annulus	2.34 ± 0.28	2.16 ± 0.26	0.19	0.11	<0.01
Sinuses of Valsalva	3.02 ± 0.39	2.90 ± 0.34	0.11	0.13	<0.01
Sinotubular junction	2.41 ± 0.32	2.44 ± 0.25	−0.03	0.15	0.43
Ascending aorta	2.31 ± 0.27	2.59 ± 0.28	−0.28	0.19	<0.01
30-39 yr					
Annulus	2.31 ± 0.29	2.15 ± 0.24	0.16	0.13	<0.01
Sinuses of Valsalva	3.20 ± 0.40	3.08 ± 0.34	0.13	0.11	<0.01
Sinotubular junction	2.48 ± 0.25	2.54 ± 0.24	−0.06	0.13	0.03
Ascending aorta	2.50 ± 0.30	2.77 ± 0.26	−0.27	0.21	<0.01
40-49 yr					
Annulus	2.28 ± 0.23	2.13 ± 0.20	0.15	0.10	<0.01
Sinuses of Valsalva	3.18 ± 0.40	3.07 ± 0.38	0.11	0.12	<0.01
Sinotubular junction	2.59 ± 0.35	2.57 ± 0.29	0.02	0.15	0.50
Ascending aorta	2.59 ± 0.30	2.85 ± 0.31	−0.27	0.19	<0.01
50-59 yr					
Annulus	2.36 ± 0.19	2.17 ± 0.16	0.19	0.13	<0.01
Sinuses of Valsalva	3.34 ± 0.28	3.19 ± 0.28	0.14	0.13	<0.01
Sinotubular junction	2.73 ± 0.24	2.64 ± 0.25	0.09	0.13	<0.01
Ascending aorta	2.90 ± 0.39	3.12 ± 0.34	−0.22	0.23	<0.01
60-69 yr					
Annulus	2.32 ± 0.21	2.12 ± 0.21	0.20	0.12	<0.01
Sinuses of Valsalva	3.42 ± 0.35	3.27 ± 0.33	0.16	0.09	<0.01
Sinotubular junction	2.83 ± 0.27	2.82 ± 0.30	0.01	0.19	0.88
Ascending aorta	3.01 ± 0.29	3.18 ± 0.33	−0.17	0.13	<0.01

*ASE* American Society of Echocardiography.

Data are expressed as mean ± SD.

^*^2005 American Society of Echocardiography chamber quantification guidelines.

^†^2010 American Society of Echocardiography pediatric measurements guidelines.

^‡^*P* values were obtained by paired *t* test.

Difference = (measurement of ASE 2005) – (measurement of Pediatric 2010).

Associations between clinical characteristics and aortic root diameters obtained with two different guidelines are presented in Table 3. Diameters of sinus of Valsalva, sinotubular junction, and ascending aorta showed significant positive correlation with age; however, aortic annulus size did not show significant relationship with age. On the other hands, aortic annulus size showed significant correlation with BSA and gender. DBP showed significant correlation with ascending aorta diameter only measured by 2005 ASE guideline.

**Table 3:** Associations between clinical characteristics and aortic root diameters measured by 2005 American society of echocardiography chamber quantification guidelines and 2010 American society of echocardiography pediatric measurements guidelines by multiple linear regression analysis

	2005 ASE ^*^			**2010 Pediatric **^**†**^		
	**β ± SE**	**R**^**2**^^**‡**^	***P***	**β ± SE**	**R**^**2**^^**‡**^	***P***
Annulus						
Age	0.002 ± 0.001	0.013	0.23	0.001 ± 0.001	0.007	0.38
BSA	0.570 ± 0.152	0.116	<0.01	0.478 ± 0.128	0.116	<0.01
Gender	0.140 ± 0.053	0.063	0.01	0.147 ± 0.044	0.094	<0.01
DBP	0.001 ± 0.002	0.002	0.59	0.001 ± 0.002	0.002	0.66
Sinuses of Valsalva						
Age	0.013 ± 0.002	0.236	<0.01	0.011 ± 0.002	0.213	<0.01
BSA	0.531 ± 0.228	0.048	0.02	0.433 ± 0.215	0.036	0.05
Gender	0.531 ± 0.228	0.102	<0.01	0.260 ± 0.074	0.102	<0.01
DBP	0.003 ± 0.003	0.009	0.30	0.002 ± 0.003	0.008	0.35
Sinotubular junction						
Age	0.013 ± 0.002	0.307	<0.01	0.011 ± 0.002	0.252	<0.01
BSA	0.373 ± 0.194	0.033	0.06	0.345 ± 0.190	0.030	0.07
Gender	0.187 ± 0.067	0.067	0.01	0.183 ± 0.066	0.068	0.01
DBP	0.002 ± 0.002	0.009	0.31	0.000 ± 0.002	0.000	0.96
Ascending aorta						
Age	0.019 ± 0.002	0.370	<0.01	0.017 ± 0.002	0.326	<0.01
BSA	0.368 ± 0.245	0.021	0.14	0.361 ± 0.239	0.021	0.13
Gender	0.051 ± 0.085	0.003	0.55	0.089 ± 0.083	0.011	0.28
DBP	0.007 ± 0.003	0.059	0.01	0.004 ± 0.003	0.023	0.12

ASE, American Society of Echocardiography; BSA, body surface area; DBP, diastolic blood pressure.

^*^2005 American Society of Echocardiography chamber quantification guidelines.

^†^2010 American Society of Echocardiography pediatric measurements guidelines.

^‡^Partial R^2^ represents explanatory power of each variable for aortic root diameter.

Table 4 shows the regression results for aortic root measurements on BSA in each age group using 2005 ASE guidelines and 2010 ASE pediatric guidelines. BSA showed good association with diameter of aortic annulus in age groups under 50 year old. This association disappeared in age over 50 year old. Ascending aorta size did not show good association with BSA.

**Table 4:** Associations between body surface area and aortic root diameters measured by 2005 American society of echocardiography chamber quantification guidelines and 2010 American society of echocardiography pediatric measurements guidelines according to age groups by linear regression analysis

	2005 ASE ^*^				**2010 Pediatric **^**†**^			
	**β**_**0**_	**β**_**1**_	**SD**	**R**^**2**^	**β**_**0**_	**β**_**1**_	**SD**	**R**^**2**^
20-29 yr								
Annulus	0.39	1.11	0.28	0.67	0.29	1.06	0.26	0.73
Sinuses of Valsalva	0.32	1.53	0.39	0.66	0.48	1.38	0.34	0.70
Sinotubular junction	0.40	1.15	0.32	0.54	0.78	0.95	0.25	0.59
Ascending aorta	1.25	0.61	0.27	0.21	1.04	0.89	0.28	0.43
30-39 yr								
Annulus	0.47	1.06	0.29	0.58	0.63	0.88	0.24	0.59
Sinuses of Valsalva	0.98	1.28	0.40	0.44	1.08	1.15	0.34	0.48
Sinotubular junction	1.30	0.68	0.25	0.33	1.26	0.74	0.24	0.39
Ascending aorta	1.01	0.86	0.30	0.36	1.71	0.61	0.26	0.24
40-49 yr								
Annulus	0.03	1.30	0.23	0.73	0.42	0.99	0.20	0.52
Sinuses of Valsalva	−0.07	1.89	0.40	0.50	0.26	1.63	0.38	0.41
Sinotubular junction	−0.09	1.56	0.35	0.44	0.49	1.20	0.29	0.38
Ascending aorta	0.86	1.00	0.30	0.25	0.85	1.16	0.31	0.32
50-59 yr								
Annulus	1.74	0.37	0.19	0.08	1.26	0.54	0.16	0.26
Sinuses of Valsalva	2.24	0.66	0.28	0.13	2.32	0.52	0.28	0.08
Sinotubular junction	1.75	0.59	0.24	0.14	1.45	0.71	0.25	0.18
Ascending aorta	1.68	0.73	0.39	0.08	2.02	0.66	0.34	0.08
60-69 yr								
Annulus	1.31	0.61	0.21	0.18	1.31	0.49	0.21	0.12
Sinuses of Valsalva	2.60	0.49	0.35	0.04	2.50	0.46	0.33	0.04
Sinotubular junction	2.25	0.35	0.27	0.04	2.81	0.01	0.30	0.00
Ascending aorta	2.74	0.17	0.29	0.01	3.03	0.10	0.33	0.00

ASE, American Society of Echocardiography.

^*^2005 American Society of Echocardiography chamber quantification guidelines.

^†^2010 American Society of Echocardiography pediatric measurements guidelines.

Based on this Table 4, one can obtain predicted mean aortic diameter with equation below. Mean aortic diameter = β_0_ + β_1_ × BSA. Then, one can calculate z score as below. Z = (measured aortic root diameter – mean aortic root diameter)/SD.

Table 5 represents intraobserver variability data of aortic root measurements as determined by the 2005 ASE guidelines and the 2010 ASE pediatric guidelines. The mean differences between two measurements were very small and statistically insignificant. Intraobserver comparison of aortic diameters measured by the 2005 ASE guidelines and 2010 ASE pediatric guidelines showed high ICCs (aortic annulus, 0.865 and 0.805; sinuses of Valsalva, 0.962 and 0.965; sinotubular junction, 0.936 and 0.940; ascending aorta, 0.948 and 0.973, respectively). The measurements by 2005 ASE guidelines and 2010 ASE pediatric guidelines showed similar intraobserver variability. Results of interobserver comparisons are shown in Table 6. Most means and SDs of the differences were larger than those of intraobserver variability.

**Table 5:** Intraobserver comparison of aortic diameters measured by two different echocardiography guidelines

	**Observation1**	**Observation2**	**Difference**	**ICC**
			Mean ± SD P *	
2005 ASE ^†^				
Annulus	2.42 ± 0.23	2.42 ± 0.19	−0.00 ± 0.11 0.95	0.87
Sinuses of Valsalva	3.24 ± 0.33	3.24 ± 0.31	−0.01 ± 0.09 0.77	0.96
Sinotubular junction	2.59 ± 0.28	2.63 ± 0.25	−0.03 ± 0.10 0.26	0.94
Ascending aorta	2.72 ± 0.39	2.73 ± 0.37	−0.02 ± 0.12 0.59	0.95
2010 ASE pediatric ^‡^				
Annulus	2.20 ± 0.21	2.25 ± 0.18	−0.05 ± 0.12 0.06	0.81
Sinuses of Valsalva	3.11 ± 0.31	3.13 ± 0.30	−0.02 ± 0.08 0.28	0.97
Sinotubular junction	2.58 ± 0.25	2.60 ± 0.26	−0.02 ± 0.09 0.41	0.94
Ascending aorta	2.95 ± 0.42	2.96 ± 0.41	−0.01 ± 0.10 0.77	0.97

*ASE* American Society of Echocardiography, *ICC* intraclass correlation coefficient.

Data are expressed as mean ± SD.

^*^P values were obtained by paired *t* test.

^†^2005 American Society of Echocardiography chamber quantification guidelines.

^‡^2010 American Society of Echocardiography pediatric measurements guidelines.

**Table 6:** Interobserver comparison of aortic diameters measured by two different echocardiography guidelines

	**Observer1**	**Observer2**	**Difference**	**ICC**
			Mean ± SD P^*^	
2005 ASE^†^				
Annulus	2.42 ± 0.19	2.44 ± 0.21	−0.02 ± 0.12 0.49	0.83
Sinuses of Valsalva	3.24 ± 0.31	3.27 ± 0.29	−0.03 ± 0.10 0.24	0.94
Sinotubular junction	2.62 ± 0.25	2.78 ± 0.34	−0.17 ± 0.29 0.02	0.45
Ascending aorta	2.73 ± 0.37	2.94 ± 0.39	−0.21 ± 0.39 0.03	0.43
2010 ASE pediatric^‡^				
Annulus	2.25 ± 0.18	2.20 ± 0.17	0.06 ± 0.17 0.16	0.47
Sinuses of Valsalva	3.13 ± 0.30	3.19 ± 0.27	−0.06 ± 0.13 0.05	0.89
Sinotubular junction	2.60 ± 0.26	2.71 ± 0.30	−0.12 ± 0.13 <0.01	0.82
Ascending aorta	2.96 ± 0.41	2.98 ± 0.40	−0.03 ± 0.21 0.61	0.87

*ASE* American Society of Echocardiography, *ICC* intraclass correlation coefficient.

Data are expressed as mean ± SD.

^*^P values were obtained by paired *t* test.

^†^2005 American Society of Echocardiography chamber quantification guidelines.

^‡^2010 American Society of Echocardiography pediatric measurements guidelines.

## Discussion

This study detailed the normal values of aortic root measurements assessed with the 2005 ASE guidelines and the 2010 ASE pediatric guidelines in a Korean population. The results demonstrate variable aortic root diameters according to the measurement guideline used.

### Background on the development of two different measurement guidelines

Measurement methods in 2005 ASE guidelines and the 2010 ASE pediatric guidelines have been developed independently with several considerations. The results in control subjects studied by Roman et al. [3] were published in era of transition from M-mode to 2D echocardiography. The techniques used for M-mode assessment were transferred to 2D echocardiography for the purpose of comparing the two techniques. Roman et al. state explicitly that "Measurements were made at end-diastole using the leading edge technique”. Subsequent studies, including important outcomes studies in Marfan patients [17,18], adopted this technique. The availability of normal controls and the publication of outcome studies based on the methods used in earlier studies may have argued for the use of this approach in the 2005 guideline paper by Lang et al. [11] On the other hand, early pediatric echocardiographic investigations measured aortic dimensions at maximum expansion (i.e., at the time of peak aortic wall stress) because of the thought that this measurement would be more predictive of the risk for aortic dissection, a relevant worry in children with aortic enlargement. Children’s Hospital Boston group suggested normal control values [19] and measurement method were developed without an awareness of the technique of Roman et al. Some important pediatric studies have utilized the Boston Childrens’/pediatric technique, including an ongoing study on Marfan syndrome [20,21]. This may account for why these two guideline documents used different methods for measuring aortic diameters.

### Different measurement results from 2005 ASE and by 2010 ASE pediatric guideline

Differences between two measurements by 2005 ASE guideline (using a leading-leading edge methodology) and 2010 ASE pediatric guideline (using an inner-inner measurement) is quite predictable because the former includes one side of aortic wall thickness, whereas later is not. Sinotubular junction is thinner than ascending aortic wall; thus difference between two methods is minimal (less than 1mm) in sinotubular junction. Measurement of sinuses of Valsalva was different about 1mm which represents aortic wall thickness.

However, another important determinant for aortic root measurement is timing of measurement during cardiac cycle. Normal histology of the aortic root shows prominent elastic lamellae in aortic wall, but it ends abruptly at the point of union with the aortic root and fibrous annulus within the sinuses of Valsalva. [22] Therefore, the change of diameter between systole and diastole is normally about 2 mm/m^2^ in the ascending aorta and results in different measurements according to cardiac cycle, as has been described previously [23,24]. Therefore, systolic distension of ascending aorta compared to diastolic phase (aortic distensibility) overwhelms the difference from aortic wall thickness in 2005 ASE guideline. On the other hand, the cycle changes of aortic annulus, sinuses of Valsalva, and sinotubular junction diameters were minimal according to the cardiac cycle because of less elastic tissue in vascular wall [25]. Distensability of ascending aorta decreased with aging process or in pathologic aorta (Takayasu arteritis, Marfan’s syndrome etc.), thus difference between two measurements should decrease according to age. Our data shows trend to decrease differences between two measurements of ascending aorta according to age.

Unifying these two measurement method can be convenient when pediatric patients grow up and move to adult clinics. However, these two different techniques are now well-accepted practices with predictive information for children and adults. If we apply the pediatric guideline to adult population, the clinical implication of measurement results is unclear because of rare clinical experience. Therefore, both methods are valuable in current era. However, clinician should keep in mind that different data present between pediatric and adult guideline when reviewing previous echocardiographic reports from pediatric echo laboratory.

Another consideration for measurement methods is communication with other modalities. This is an important aspect, as aortic values derived from CT imaging and MRI are based mainly on the inner edge convention, and in the current era of multimodality assessment, comparison between different methods of imaging is mandatory in attempting to facilitate communicability, data exchange, and patient monitoring among different labs. Therefore, relevance in 2010 ASE pediatric guideline (using an inner-inner measurement) is better comparison to other imaging methods and it can make less confusion with the ascending aorta size which clearly expands in systole in medical communication.

### Influence of age and BSA in aortic root size

The mean diameters of sinuses of Valsalva, sinotubular junction, and ascending aorta increased as age increased in accordance with previous studies [9,26]. However, aortic annular diameter did not appear to vary with age. Rather, aortic annulus diameter showed good correlation with BSA.

Association with age and ascending aortic diameter is reported in previous study [26,27]. Aortic wall of ascending aorta has much elastic tissue. Pressure loading continues during whole lifespan. Therefore, dilation of aorta increases with aging. Diameter of sinus of Valsalva also increase with age in our study and other previous study [27]; however the growth with age is relatively slower than ascending aorta. Because fibrous tissue is more abundant than elastic tissue in sinus of Salsalva and dilation of sinus of Valsalva is possibly slower than ascending aorta. Blood pressure was correlated with ascending aorta diameter using 2005 ASE guideline. Hypertension is an important risk factor for aortic dissection and aortic aneurysm; however association between ascending aortic diameter and DBP was reported small in Framingham Heart Study group [28]. Our study also shows similar results although we only included the “clinical normal” subjects and impact of blood pressure was possibly denudated in our study.

Aortic annulus was strongly associated BSA especially in younger aged population. Predictor of aortic valve diameter with body surface area has been reported in surgical experience [29]. Aortic annulus diameter is associated with body growth in pediatric patients. Larger BSA is associated larger ventricular volume in normal population [30] and normal growth of aortic annulus as cardiac-vascular junction is possibly associated with cardiac size.

Interestingly, this association decreased in old aged group (more than 50 year-old). The cause of this result is uncertain, but similar result was reported in a previous study and it has been speculated that in middle and older age body weight (and hence BSA) may deviate progressively from that during the years when aortic size was programmed, and that subclinical degenerative processes altering aortic composition and distensibility may occur in some but not other persons [9]. Therefore, detecting abnormal aortic dilation should be determined based on individual age, BSA, and gender and z score with regression equation for normal population can be helpful.

Detecting abnormal dilation of aortic root size is important in vascular disease. Aortic root size is adjusted for age and body size, and a Z-score ≥2 is used as the cut-off value. Therefore, providing a normal value according to age is important as a reference and the method for aortic root measurement should be standardized. Furthermore, when diagnosis is made in childhood, regular follow-up for aortic root dilatation is recommended through childhood to adult. In clinics, physicians should identify the measurement method in interpreting the change of aortic root diameters during follow up of these patients. If there is a sudden change of aortic diameter, direct review of echocardiographic images will be helpful. Although the 2005 ASE guidelines recommend measurements of the aortic root from the leading edge to the leading edge, some institutions favor the inner edge to inner edge technique in the measurement of aortic root diameters of adult patients [11]. These measurement methods should be specified in the report because different results by different measurement methods can influence the assessment of patient’s prognosis.

### Limitation

The number of cases is certainly sufficient for the measurement analysis and comparison between the two methods, however considerations and correlations regarding the influence of all biological variables are limited considering numbers of cases in each decade. As well, gender has not been considered due to limited number of cases in each decade as well as this study is not aimed at ratiometric and allometric analysis.

## Conclusion

We evaluated the detailed aortic root diameters with TTE using two different measurement guidelines. Aortic root measurement values, their associations with clinical characteristics, and reliability and validity of measurement with TTE can be influenced by measurement methods and locations within aortic root. Age and BSA influence to aortic root size, however association is different in each site of aortic root. Therefore, physicians should consider not only measurement method used but also different decades of age, BSA, wight, height and gender when assessing the aortic root diameters and their changes during follow-up of patients.

## Competing interests

The authors declare that they have no competing interests.

## Authors’ contributions

MKS and SC wrote the manuscript and performed the data analysis. SC and DK made study concept and design and approved final manuscript. JHK and HJL performed echocardiography and data collection. SP, JC, SL, SWP, and JKO made critical comments for this work.

All authors read and approved the final manuscript.
